# Effect of preoperative chemotherapy on the histopathological classification of gastric cancer

**DOI:** 10.1007/s10120-023-01442-w

**Published:** 2023-11-10

**Authors:** I. A. Caspers, H. D. Biesma, K. Wiklund, F. Pontén, P. Lind, M. Nordsmark, K. Sikorska, E. Meershoek-KleinKranenbarg, H. H. Hartgrink, C. J. H. van de Velde, J. W. van Sandick, M. Verheij, A. Cats, N. C. T. van Grieken

**Affiliations:** 1 Department of Gastrointestinal Oncology, Netherlands Cancer Institute, Antoni Van Leeuwenhoek, Amsterdam, The Netherlands; 2 Department of Pathology, Cancer Center Amsterdam, Amsterdam University Medical Center, De Boelelaan 1117, 1081HV Amsterdam, the Netherlands; 3 Department of Oncology and Pathology, Karolinska Institute, Stockholm, Sweden; 4 Department of Immunology, Genetics and Pathology, Rudbeck Laboratory, Uppsala University, Uppsala, Sweden; 5 Department of Oncology, Aarhus University Hospital, Aarhus, Denmark; 6 Department of Biometrics, Netherlands Cancer Institute, Antoni Van Leeuwenhoek, Amsterdam, The Netherlands; 7 Department of Surgical Oncology, Leiden University Medical Center, Leiden, The Netherlands; 8 Department of Surgical Oncology, Netherlands Cancer Institute, Antoni Van Leeuwenhoek, Amsterdam, The Netherlands; 9 Department of Radiation Oncology, Radboud University Medical Center, Nijmegen, The Netherlands; 10 Department of Radiation Oncology, Netherlands Cancer Institute, Antoni Van Leeuwenhoek, Amsterdam, The Netherlands

**Keywords:** Gastric cancer, Lauren classification, Histological subtype, Concordance

## Abstract

**Background:**

In the era of individualized gastric cancer (GC) treatment, accurate determination of histological subtype becomes increasingly relevant. As yet, it is unclear whether preoperative chemotherapy may affect the histological subtype. The aim of this study was to assess concordance in histological subtype between pretreatment biopsies and surgical resection specimens before and after the introduction of perioperative treatment.

**Methods:**

Histological subtype was centrally determined in paired GC biopsies and surgical resection specimens of patients treated with either surgery alone (SA) in the Dutch D1/D2 study or with preoperative chemotherapy (CT) in the CRITICS trial. The histological subtype as determined in the resection specimen was considered the gold standard. Concordance rates and sensitivity and specificity of intestinal, diffuse, mixed, and “other” subtypes of GC were analyzed.

**Results:**

In total, 105 and 515 pairs of GC biopsies and resection specimens of patients treated in the SA and CT cohorts, respectively, were included. Overall concordance in the histological subtype was 72% in the SA and 74% in the CT cohort and substantially higher in the diffuse subtype (83% and 86%) compared to the intestinal (70% and 74%), mixed (21% and 33%) and “other” subtypes (54% and 54%). In the SA cohort, sensitivities and specificities were 0.88 and 0.71 in the intestinal, 0.67 and 0.93 in the diffuse, 0.20 and 0.98 in the mixed, and 0.50 and 0.93 in the “other” subtypes, respectively.

**Conclusion:**

Our results suggest that accurate determination of histological subtype on gastric cancer biopsies is suboptimal but that the impact of preoperative chemotherapy on histological subtype is negligible.

## Introduction

Gastric cancer (GC) is morphologically, biologically, and clinically a highly heterogeneous disease [1]. In 1965, Lauren described two major histopathological subtypes, intestinal type, and diffuse type GC, that differ in both histopathological patterns, clinicopathological characteristics, response to systemic treatment, and prognosis [2–4]. The intestinal subtype is recognized by gland-like structures, while the diffuse subtype is characterized by a poorly cohesive growth pattern, often in the presence of signet ring cells [2]. In the mixed subtype, described by the WHO classification in 2000, components of both subtypes can be recognized [5].

The current standard treatment for non-metastatic, resectable GC in most European countries consists of perioperative chemotherapy [6]. To date, no distinction is being made in the choice of treatment between Lauren’s histological subtypes, despite the fact that the intestinal subtype is associated with a better prognosis and better response to chemotherapy compared to the diffuse subtype [3, 7, 8]. The FLOT4-AIO trial showed a (near-)complete histological response and overall survival benefit of the 5-fluorouracil, leucovorin, oxaliplatin, and docetaxel (FLOT) chemotherapy regimen over the epirubicin, cisplatin and capecitabine (ECX) chemotherapy. In subgroup analyses of this trial, however, this (near-)complete histological response and overall survival benefit could not be demonstrated in the group of patients with diffuse type GC [9, 10]. As a consequence, it seems appropriate to address intestinal and diffuse subtypes as distinct tumor entities.

Fortunately, the histological subtype is a commonly used stratification factor in randomized clinical trials [9, 11]. In the era of individualized GC treatment, accurate histological subtyping on pretreatment biopsies may become increasingly relevant for treatment choices. For practical reasons, the surgical resection specimen is generally considered the preferred method for evaluating tumors since it enables a more precise and extensive assessment of the histological subtype. Several studies have investigated the concordance of the GC histological subtype between pretreatment biopsies and surgical resection specimens and reported an overall concordance of 65–75% [12–14], with the highest concordance of 90% for the diffuse histological subtype [15].

These studies, however, included only patients treated with surgery alone.[12–15]. The impact of the current standard of care treatment with preoperative chemotherapy on histological subtyping is therefore yet unknown. The surgical resection specimen is typically regarded as the gold standard because it provides the pathologist with a large amount of tumor tissue, allowing for a more representative and accurate evaluation of the tumor characteristics and intratumor heterogeneity. This might, however, be unsuitable in the preoperatively treated population, as the treatment may affect tumor morphology and composition. Currently, it is difficult to determine whether disagreement in histological subtype determined on pretreatment biopsies and resection specimens indicates true discrepancies or actually displays a chemotherapeutic effect. For instance, a mixed-type tumor might be mistaken for a diffuse tumor in the resection specimen after a pathological complete response of its intestinal component [16]. Since the histological subtype is often used for stratification in clinical trials, the question whether the histological subtype determined on pretreatment biopsies and resection specimens can be used interchangeably in the preoperatively treated population becomes increasingly relevant.

The aim of this study was therefore to determine the effect of preoperative systemic treatment on the GC histological subtype. We present the concordance in GC histological subtype between paired pretreatment biopsies and surgical resection specimens from patients treated with either surgery alone as part of the Dutch D1/D2 study [17], or with perioperative treatment as part of the CRITICS trial [11].

## Methods

### Patients

All patients of the Dutch D1/D2 study and CRITICS trial of whom pathology slides of both pretreatment biopsy and resection specimen were available for histopathological review, were included in this analysis in either the surgery alone (D1/D2 study) or chemotherapy (CRITICS trial) cohort. In the chemotherapy cohort, patients with a histologically complete response were excluded as their resection specimens did not contain tumor cells, thus hindering the assessment of the histological subtype.

In the Dutch D1/D2 study (1989–1993), 1078 patients with histologically proven resectable gastric cancer were randomized between gastric cancer resection with either a D1 or D2 lymphadenectomy [17]. A gastrectomy was performed in 711 of the included patients. None of these patients underwent any preoperative treatment.

In the CRITICS trial (2009–2015, NCT00407186), 788 patients with resectable gastric cancer (stage Ib-IVa, AJCC 6th edition TNM) were upfront randomized between either perioperative chemotherapy and surgery or a combination of preoperative chemotherapy, surgery, and postoperative chemoradiotherapy [18]. Preoperative chemotherapy consisted of three cycles of epirubicin, oxaliplatin or cisplatin, and capecitabine. In total, 741 of the included patients proceeded to surgery. Lauren classification at baseline was a stratification factor for inclusion in the CRITICS trial [11]. Primary outcomes of the D1/D2 and CRITICS trials have been published previously [11, 17, 19].

### Histopathology review

A central review of hematoxylin-and-eosin (H&E) stained pathology slides of pretreatment biopsies and resection specimens was carried out by an expert gastrointestinal pathologist (NCTvG). Histopathological tumor types were classified according to Smyth et al. as intestinal type, diffuse type, mixed type, or “other” type [1]. “Other” histological subtypes include tumors that have been recognized by the WHO classification but do not fit in Lauren classification (e.g., mucinous type adenocarcinoma, undifferentiated carcinoma, and carcinoma with lymphoid stroma).

To assess factors, other than chemotherapy, that are potentially associated with discordance in histological subtype, the number of (tumor-positive) biopsies was counted on pathology slides of pretreatment biopsies in the D1/D2 study. Also, tumor regression grade according to Mandard (TRG, in which TRG1 indicates a complete response and TRG5 indicates no response) was assessed [20]. Since tumor regression-like changes, such as fibrosis and stromal reactions, have been described even in patients treated with surgery alone, TRG was determined both in the chemotherapy and surgery-alone cohorts to evaluate potential bias [21].

### Statistical analysis

Concordance between paired pretreatment biopsies and resection specimens was calculated for all histological subtypes combined and for each histological subtype separately in both cohorts. Concordance rates were calculated as the percentage of identical histological subtype on pretreatment biopsy and resection specimen. Sensitivity and specificity were calculated for each histological subtype in the surgery-alone cohort. For this purpose, histological subtype as determined on the resection specimen was considered the gold standard. Concordance rates for patient subgroups (e.g., categorized by sex and UICC 8th edition TNM stages) were presented as percentages with 95% confidence intervals. Associations between concordance and other histopathological parameters (e.g., number of biopsies and TRG) were tested using the Mann–Whitney *U* test for numerical variables and the Chi-Square test, or Fisher exact test when appropriate, for categorical variables. Statistical significance was set at *p* < 0.05. All analyses were performed using R software version 4.0.3.

## Results

### Surgery-alone cohort

Histological subtype of GC was determined on pretreatment biopsies of 105 patients, resulting in 60 (57%) intestinal type, 29 (28%) diffuse type, 3 (3%) mixed type, and 13 (12%) “other” type GCs. In the paired resection specimens, GCs were classified as an intestinal subtype in 50 (48%) patients, diffuse type in 36 (34%) patients, mixed type in 5 (5%) patients, and “other” subtypes in 14 (13%) patients. Overall concordance between pretreatment biopsies and resection specimens was 72% (i.e., in 76 of 105 patients). Table 1 shows the agreement between pretreatment biopsies and resection specimens for each histological subtype. Concordance was 74% in the intestinal type, 83% in the diffuse subtype, 33% in the mixed subtype, and 54% in the “other” subtype.

**Table 1:** Concordance between histological subtype in pretreatment biopsies and resection specimens in gastric cancer patients in the surgery alone cohort (*n* = 105). Overall concordance: 72%

	Intestinal *biopsy (n* = *60)*	Diffuse *biopsy (n* = *29)*	Mixed *biopsy (n* = *3)*	Other *biopsy (n* = *13)*
Intestinal *resection (n* = *50)*	44 (74%)	1 (3%)	1 (33%)	4 (31%)
Diffuse *resection (n* = *36)*	9 (15%)	24 (83%)	1 (33%)	2 (15%)
Mixed *resection (n* = *5)*	2 (3%)	2 (7%)	1 (33%)	0
Other *Resection (n* = *14)*	5 (8%)	2 (7%)	0	7 (54%)

Sensitivity and specificity were 0.88 and 0.71 for the intestinal subtype, 0.67 and 0.93 for the diffuse subtype, 0.20 and 0.98 for the mixed subtype, and 0.50 and 0.93 for the “other” subtypes, respectively.

### Chemotherapy cohort

Histopathological subtype was determined in 515 paired pretreatment biopsies and resection specimens. The intestinal, diffuse, mixed, and “other” histological subtypes were found in 216 (42%), 236 (46%), 24 (5%), and 39 (7%) pretreatment biopsies and in 177 (34%), 241 (47%), 25 (5%) and 72 (14%) resection specimens, respectively. Overall concordance was found in 381 (74%) cases. Table 2 shows the concordance between pretreatment biopsies and resection specimens for each histological subtype. The highest concordance rate was observed in the diffuse subtype with similarity in 86% of cases. In intestinal, mixed, and “other” subtypes, this was 70%, 21%, and 54%, respectively.

**Table 2:** Concordance between histological subtype in pretreatment biopsies and resection specimens in gastric cancer patients in the chemotherapy cohort. (*n* = 515). Overall concordance: 74%

	Intestinal *biopsy (n* = *216)*	Diffuse *biopsy (n* = *236)*	Mixed *biopsy (n* = *24)*	Other *biopsy (n* = *39)*
Intestinal *resection (n* = *177)*	151 (70%)	14 (6%)	5 (21%)	7 (18%)
Diffuse *Resection (n* = *241)*	21 (9%)	203 (86%)	8 (33%)	9 (23%)
Mixed *Resection (n* = *25)*	12 (6%)	6 (3%)	5 (21%)	2 (5%)
Other *Resection (n* = *72)*	32 (15%)	13 (5%)	6 (25%)	21 (54%)

Table 3 shows that the number of pretreatment biopsies did not influence the degree of concordance in the surgery-alone cohort, with similar numbers of obtained biopsies in concordant and discordant pairs (6 [4–8] and 6.5 [5–8]; *p* = 0.91), respectively. Likewise, the number of tumor-positive biopsies was equal between concordant and discordant cases (3 [2–4] and 3 [2–4]; *p* = 0.82), respectively.

**Table 3:** Association between number of (tumor-positive) pretreatment biopsies and concordance of histological subtype in the D1/D2 study

	Concordant *(n* = *76)*	Discordant *(n* = *29)*	*p value*
Total number of biopsies *Median (IQR)*	6 (4 – 8)	6.5 (5 – 8)	*0.91*
Tumor-positive biopsies *Median (IQR)*	3 (2 – 4)	3 (2 – 4)	*0.82*

**Fig. 1:**
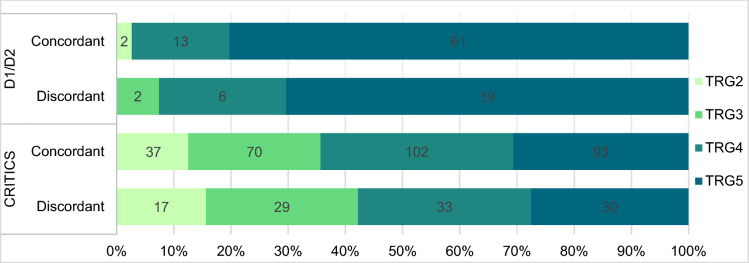
Tumor regression grade (TRG) assessed on resection specimens of patients included in the D1/D2 study (*n* = 103) and CRITICS trial (*n* = 411)

Tumor regression grade (TRG) was assessed in 103 (98%) resection specimens in the surgery-alone cohort. As seen in Fig. 1, 98% (101 of 103 tumors) showed minimal or no tumor response (TRG4-5). Interestingly, two cases (2%) with the near-complete response (TRG2) were identified, even though preoperative chemotherapy had not been administered in these patients. It is noteworthy that both these cases involved tumors that were limited to the mucosa and showed fibrotic changes in the underlying submucosal layer.

In the chemotherapy cohort, TRG was assessed in 411 (80%) resection specimens. In 63% of the tumors, minimal to no tumor regression (TRG4-5) was seen. Near-complete response (TRG2) was seen in 13% of cases. Tumor regression grade was not significantly associated with concordance of histological subtype. (*p* = 0.646).

**Fig. 2:**
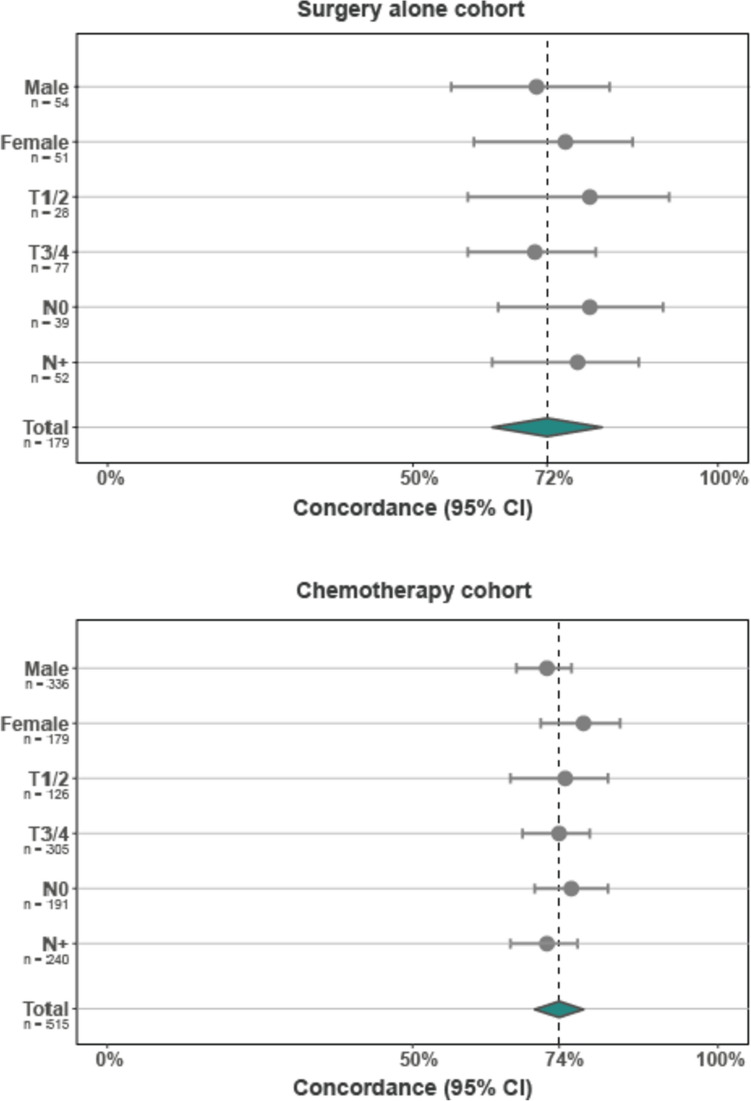
Concordance per patient subgroup. CI confidence interval

Overall, concordance rates of histological subtypes between biopsy samples and resection specimens in the surgery alone and the chemotherapy cohorts were similar (72% and 74%, *p* = 0.766) Concordance rates did not differ significantly among subgroups of patients categorized by sex, pT stage, and pN stage (Fig. 2).

## Discussion

In this study, involving two large cohorts of gastric cancer patients treated with either surgery alone (D1/D2 study) or with surgery and perioperative treatment (CRITICS trial), the concordance in histological subtype between pretreatment biopsies and resection specimens was investigated. The histological subtype is a commonly used stratification factor for clinical trials involving GC patients. However, the impact of preoperative systemic treatment on the different histological subtypes of GC is yet undetermined. To our knowledge, this is the first study to evaluate concordance between biopsies and resection specimens in patients treated with preoperative chemotherapy. We found an overall concordance in histological subtype of 72% in the surgery alone cohort and 74% in the chemotherapy cohort. This similarity in concordance rates indicates that the impact of systemic therapy on the histological subtype is negligible and that both pretreatment biopsies and surgical resection specimens can be used for the determination of the histological subtype in gastric cancer patients.

Histological subtypes of gastric cancer are widely recognized to represent distinct biological and clinical entities with regard to treatment response and prognosis, and can therefore be useful as prognostic markers [9, 16, 22]. The potential utility of histological subtype as a predictive marker to guide therapeutic decisions, however, remains a matter of debate. Although new therapies with corresponding molecular markers, such as HER2 for trastuzumab and PD-L1 expression for immune checkpoint inhibitors, show promise, they only apply for approximately 22% and 16% of patients, respectively [23, 24]. This means that personalized treatment options are as yet unavailable for the majority of GC patients. Meanwhile, the Lauren classification is readily available at low clinical costs. Therefore, accurate determination and stratification by histological subtype in clinical trials will remain crucial until novel markers arise.

Historically, we relied on pretreatment biopsies for stratification in clinical trials since the impact of chemotherapy on histological subtype was yet unknown. Our study, however, shows that chemotherapy has a negligible impact on histological subtype. Clinical trials in which patients are postoperatively randomized between treatment arms, for example, the VESTIGE trial that randomizes between chemotherapy and immunotherapy after surgical resection, could therefore stratify patients for histological subtype assessed on either the biopsy or resection specimen [25]. This could be relevant in case the biopsy specimen is not available. Similarly, studies investigating novel treatment modalities in patients with metachronous metastases can use histological subtype as assessed on the resection specimen for stratification. Since more material is available for evaluation in the resection specimen and it provides more insight into intratumor heterogeneity, we believe that histological subtype assessment for stratification in clinical trials does not have to be confined to pretreatment biopsies.

Our results are in line with those of previous studies that reported an overall concordance in histopathological subtype between biopsies and resection specimens of 65–75% [12–14]. A substantially higher concordance rate of 83–86% was seen in the diffuse subtype. This is in line with the study of Piessen et al., which showed a higher concordance of 90% in signet ring cell carcinomas [15]. As signet ring cells are often present in diffuse type GC, it is possible that the presence of signet ring cells also has contributed to the higher level of concordance in diffuse type, compared to the intestinal type in our study. In the mixed type, the limited amount of tissue available in pretreatment biopsies may have hampered the accurate distinction of this phenotype from the intestinal and diffuse subtypes, consequently resulting in low concordance. This finding is consistent with Flucke et al. who showed similar concordance rates of 79% in intestinal, 86% in diffuse, and 56% in mixed histological subtypes in a German cohort of 100 primary treatment-naive GC patients [12]. Likewise, a Chinese study with 116 pairs of biopsy and surgery samples reported that most discrepancies in histological subtyping could be attributed to the mixed subtype [14]. Taken together, these overall levels of concordances are suboptimal as the histological subtype is a commonly used stratification factor in clinical trials﻿.

The number of (tumor-positive) biopsies and the tumor regression grade did not significantly impact the concordance of the histological subtype in our study. It is noteworthy that two tumors in the surgery-alone cohort exhibited features associated with near-complete tumor regression, despite not having undergone any preoperative treatment. This phenomenon of tumor regression-like changes in treatment-naïve GCs has previously been reported in a post-hoc analysis of the MAGIC trial, where 3 patients treated with surgery alone showed a near-complete pathological response as well [21]. Interestingly, both tumors in our study were restricted to the mucosal layer and showed fibrotic tumor regression-like changes in the submucosal layer. These findings may highlight the challenges that can be encountered in accurately assessing tumor regression in early-stage GCs.

It is noteworthy that several clinical trials using the Lauren classification assessed on pretreatment biopsies as a stratification factor reported that in approximately 30% of gastric cancers the histological subtype was not known.[9, 11]. Since the histological subtype of all included pretreatment biopsies for central review in our study could be determined, we believe this is most likely a matter of underreporting rather than of infeasibility to determine the histological subtype. As histological subtypes represent distinct biological and clinical subgroups, we would strongly recommend the (central) evaluation of histological subtype on pretreatment biopsies in clinical trials.

Nonetheless, it is possible that “other” histological subtypes that do not fit in Lauren classification [1] may have contributed to the high proportion of tumors of unknown Lauren classification in several clinical trials. The “other” histological subtype was first mentioned as a distinct category by Smyth et al. in 2020 and was diagnosed in 12% and 8% of cases in the D1/D2 study and CRITICS trial. Flucke et al. showed a higher concordance rate between biopsies and resection specimens using the more comprehensive WHO classification (distinguishing, e.g., the mucinous subtype) compared to the Lauren classification (84% vs. 74%, respectively)[12]. It supports the notion that concordance rates potentially improve with recognition and scoring of the “other” subtype. Further recognition of the “other” histological subtype should therefore be encouraged.

A limitation of this study is that interobserver agreement in GC histological subtyping could not be analyzed due to the design with central pathology review in the D1/D2 study and CRITICS trial. Interobserver variation could affect concordance rates. In previous studies, interobserver agreement varied between 70 and 90% [13, 26, 27]. Concordance rates in the surgery alone cohort, however, are similar to previous studies [12–15]. It is also important to acknowledge that the preferred chemotherapeutic regimen has shifted from an anthracycline-based treatment (as used in the CRITICS trial) to a taxane-based treatment following the publication of the results of the FLOT4-AIO trial [9]. Whether taxane-based treatment could potentially affect histological subtype remains therefore unclear.

In conclusion, accurate histological subtype determination on biopsies of gastric cancer is fairly good for diffuse-type GC, but suboptimal for intestinal GC and even less optimal in the mixed and “other” subtypes. We do however demonstrate that the impact of anthracycline-based preoperative chemotherapy on histological subtype in resection specimens is negligible. We propose that the assessment of histological subtype in clinical trials should not necessarily be confined to pretreatment biopsies, but can also be determined on resection specimens after preoperative chemotherapy.
